# Exploring antibiotic resistance load in paddy-upland rotation fields amended with commercial organic and chemical/slow release fertilizer

**DOI:** 10.3389/fmicb.2023.1184238

**Published:** 2023-04-14

**Authors:** Bingjun Han, Shizhou Shen, Fengxia Yang, Xiaolong Wang, Wenxuan Gao, Keqiang Zhang

**Affiliations:** ^1^Agro-Environmental Protection Institute, Ministry of Agriculture and Rural Affairs, Tianjin, China; ^2^Dali, Yunnan, Agro-Ecosystem, National Observation and Research Station, Dali, China; ^3^School of Environmental Science and Engineering, Nankai University, Tianjin, China

**Keywords:** antibiotic resistance genes, fertilization modes, distribution patterns, vertical profile, paddy-upland rotation soil

## Abstract

Agricultural fertilization caused the dissemination of antibiotic resistance genes (ARGs) in agro-ecological environment, which poses a global threat to crop-food safety and human health. However, few studies are known about the influence of different agricultural fertilization modes on antibiotic resistome in the paddy-upland rotation soils. Therefore, we conducted a field experiment to compare the effect of different fertilization (chemical fertilizer, slow release fertilizer and commercial organic fertilizer replacement at various rates) on soil antibiotic resistome in paddy-upland rotation fields. Results revealed that a total of 100 ARG subtypes and 9 mobile genetic elements (MGEs) occurred in paddy-upland rotation soil, among which MDR-ARGs, MLSB-ARGs and *tet*-ARGs were the dominant resistance determinants. Long-term agricultural fertilization remarkably facilitated the vertical accumulation of ARGs, in particular that *bla*_ampC_ and *tet*O in relative abundance showed significant enrichment with increasing depth. It’s worth noting that slow release fertilizer significantly increased soil ARGs, when comparable to manure with 20% replacing amount, but chemical fertilizer had only slight impact on soil ARGs. Fertilization modes affected soil microbial communities, mainly concentrated in the surface layer, while the proportion of *Proteobacteria* with the highest abundance decreased gradually with increasing depth. Furthermore, microbial community and MGEs were further proved to be essential factors in regulating the variability of ARGs of different fertilization modes by structural equation model, and had strong direct influence (*λ* = 0.61, *p* < 0.05; *λ* = 0. 55, *p* < 0.01). The results provided scientific guidance for reducing the spreading risk of ARGs and control ARG dissemination in agricultural fertilization.

## 1. Introduction

The overuse of antibiotics accelerates the spread of antibiotic resistance genes (ARGs) among bacteria, thereby reducing the effectiveness of antibiotic therapy (Cohen et al., 2017). The proliferation of ARGs has been a major problem of antibiotic contamination in the environment, which may lead to the emergence of multidrug-resistant human pathogens and ultimately reduce the efficiency of antibiotic therapy (Mahnert et al., 2019; Tiedje et al., 2019). With the rapid development of large-scale breeding industry, the problem of ARGs contamination caused by antibiotic abuse is becoming increasingly prominent (Rahman et al., 2018). The farmland application of livestock manure can amend the soil fertility and ensure sustainable soil productivity, which has a long agricultural history, and is being vigorously promoted due to the rising cost of fertilizer as well as the necessity of unwanted wastes disposal (Zhen et al., 2014). Currently, returning manure to the field is an effective way of livestock manure resource utilization, which could realize the circulation of agricultural planting and breeding resources (Xie et al., 2018; Zhi et al., 2022).

However, ARGs inevitably transfers horizontally into the farmland along with fertilization, causing serious pollution to soil environment (Zhu D. et al., 2019; Han et al., 2021). Several studies have examined the effect of manure amendment on the fate and migration of ARGs in soil (Duan et al., 2019; Yang et al., 2020; Liu et al., 2021), where the use of manure containing residual antibiotics generally increased soil ARGs. Gu et al. (2020) found that application of pig manure, chicken manure and cow manure increased soil ARG abundances in soil by 36, 30 and 11 times, respectively, compared with the control soil. Liu et al. (2021) found that the abundance and diversity of ARGs showed an upward trend in farmland soils with repeated and continuous application of livestock manure. Obviously, manure application significantly increased the diversity and abundance of ARGs in soil, and also markedly changed the composition of bacteria related to ARGs profiles (Pu et al., 2020; Barrios et al., 2021; Guo et al., 2022). ARGs-resistant bacteria contained in the soil environment may enter the plant through horizontal transfer from endophytes, and transfer exogenous ARGs to the plant endophytic system (Guron et al., 2019; Chen et al., 2019b). A large number of ARGs and a variety of related integrons have been found in fresh fruits and vegetables, increasing the risk of transmission from ARGs to humans through the food chain (Zhang et al., 2019).

The accumulation and propagation of ARGs in soil were significantly affected by different fertilization patterns. Studies have shown that the application of chemical fertilizer and organic fertilizer can increase the level of ARGs compared with the soil without fertilization (Xu et al., 2019; Wang F. H. et al., 2020). In particular, the application of livestock manure can significantly increase the diversity and abundance of ARGs in soil, resulting in the improvement of microbial resistance level in soil environment (Gu et al., 2020; Yang et al., 2020; Han et al., 2021). In order to achieve “reducing fertilizer and controlling pollution,” it is necessary to optimize fertilization mode and structure to improve the use efficiency of agricultural fertilizer and accelerate the construction of ecological circular agriculture (Zhou et al., 2019a; Chen et al., 2021; Sanz et al., 2021; Xu F. et al., 2021). However, there are few studies on the distribution pattern and vertical migration of ARGs in paddy-upland rotation fields with unique habitat, especially under different fertilization modes (Lin et al., 2016; Rahman et al., 2018; Lin et al., 2022). The results are helpful to clarify the occurrence characteristics of soil ARGs pollution spectrum under different fertilization modes, further understand the effects of different fertilization modes on farmland ecosystem, and explore the optimal fertilization mode.

In this study, the paddy-upland rotation fields in Erhai River basin of Yunnan Province were selected for research, where were set up a plot experiment (chemical fertilizer, substitution model of different amount of livestock manure, slow release fertilizer). High throughput-quantitative PCR (HT-qPCR) combined with real-time quantification PCR (qPCR) were used to explore the occurrence profiles and vertical migration patterns of ARGs in paddy-upland rotation fields under different fertilization modes. Meanwhile, we also further studied microbial communities and environmental factors, and their contribution on ARG shift in paddy-upland rotation fields, further aiming to find an appropriate agricultural fertilization to mitigate and control the spread of ARGs in farmland. This work would provide a reference for the pollution control and risk assessment of ARGs in paddy-upland rotation soil.

## 2. Materials and methods

### 2.1. Site description and sample collection

The field experiment 8 years’ fertilization was conducted at an experimental station located in Dali City (100°10′E, 25°53′N), Yunnan Province, China. The experimental plot has maintained a fixed paddy-upland rotation mode of the rice-garlic and rice-broad bean rotation cycles. The rice used was *Oryza sativa* L. (japonica hybrid early-maturing varieties), the garlic planted was *Allium sativum* L (medium-maturing varieties), and the broad bean is *Vicia faba* L (medium-maturing varieties), which are highly suitable for planting in Yunnan Province. Initial physical and chemical properties (mean values) of soil in experimental area were as follows: total nitrogen (TN), 3.3 g/kg; total phosphorus (TP), 0.97 g/kg; total potassium, 19.3 g/kg; pH, 7.1; organic matter (OM), 57.3 g/kg.

The experimental field was designed in random block design with 8 treatments: no fertilization (CK); conventional fertilizer treatment (CF); conventional fertilizer reduction 20% treatment (T1); conventional fertilization replaced by 100% organic fertilizer replacement in terms of nitrogen (T2); conventional fertilization replaced by 100% organic fertilizer replacement in terms of phosphorus (T3); conventional fertilization replaced by 100% organic fertilizer replacement in terms of nitrogen under considering 25% organic fertilizer mineralization rate (T4); conventional fertilization replaced by 100% organic fertilizer replacement in terms of phosphorus under considering 25% organic fertilizer mineralization rate (T5); slow release fertilizer instead of chemical fertilizer (T6). Fertilizer application amount of each treatment is shown in Supplementary Table S1. The commercial organic fertilizer used in this experiment consists of 80% cow manure and 20% other animal manure. There were three plots for each treatment, and a total of 24 plots for 8 fertilization modes. The area of each experimental plot is 30 m^2^ (6 m × 5 m), and the ridge of field between plots was built with cement (the width is 0.24 m, the height is 0.20 m, and 1 m is built below the ground surface). Soil samples were collected from different depths of 0–20 cm, 20–50 cm and 50–80 cm after garlic harvest in May. Each plot was sampled at 5 points and mixed evenly, and then about 1 kg of the samples were placed in the refrigerator at −20°C by quartering method for DNA extraction.

### 2.2. DNA extraction

Soil samples DNA was extracted from 0.5 g (dry weight) soil samples collected at different soil depths using FastDNA® Soil Rotation kit (MP Biomedicals, LLC, Santa Ana, United States) according to the manufacturer’s protocol. The concentration and purity of DNA extracted from soil samples were measured on a NanoVue Plus spectrophotometer (GE Healthcare, United States) and the concentration of ARGs were recalculated based on the weight of the soil used for DNA extraction. Finally, DNA samples extracted from soils with different fertilization modes were stored at−20°C for subsequent measurement.

### 2.3. High-throughput quantitative PCR

High-throughput quantitative PCR (HT-qPCR) reactions adopted Wafergen SmartChip Real-time PCR system (MicroAnaly Genetech Co., Ltd., China), which qualified 134 ARGs, 7 transpoase genes, 2 integrase genes and 16S rRNA. Details of the primers used in this study are listed in Supplementary Table S2, and the design of the primers used followed previous studies (Zhu et al., 2013; Wang et al., 2016). The reaction system contained 2 ng/μL DNA template, 500 nM each primer, 1 × LightCycler 480 SYBR Green I Master mix and 0.1 mg/ml albumin from bovine serum. The procedure steps were set as follows: initial denaturation at 95°C for 10 min, followed by 40 cycles, including denaturation at 95°C for 30 s, annealing at 60°C for 30 s (Chen et al., 2017; Han et al., 2018).

### 2.4. Quantification detection of ARGs

Real-time quantification PCR (qPCR) reactions were conducted on a 7,500 Real-Time PCR System (Applied Biological) using the DNA/RNA/RNase-free 96-well reaction plates. The total abundance of bacteria was evaluated by analyzing 16S rRNA genes of bacteria, and the variation of ARGs abundance caused by differences in background bacterial levels and DNA extraction efficiency was corrected. The qPCR reaction mixtures (20 μl) consists of 6.8 μl RNAase-free water, 0.4 μl ROX Reference DyeII, 10 μl SYBR® Premix Ex Taq TM II (Tli RNase H Plus, Takara), 0.4 μl each primer and 2 μl DNA extract with a suitable concentration or standard plasmid. The qPCR amplification conditions were set as follows: initial denaturation (95°C, 30 s), then 40 cycles of 5 s at 95°C and 34 s at 60°C. Melting curves were analyzed at temperatures ranging from 60°C to 95°C, with 1°C/read to ensure specificity, and the method of calibration curves for each target gene was shown in previous studies (Yang et al., 2019). Each DNA sample was amplified in three replicates, and RNAase-free water was used as a negative control, a threshold cycle of 31 was used as the detection limit (Ouyang et al., 2015; Xie et al., 2016). The specificity was detected by melting curve, while the fold change, relative abundance of each target gene and the normalized copy numbers of ARGs per bacterial cell (normalized abundance) were calculated according to previous studies (Su et al., 2015; Chen et al., 2016; Wang et al., 2017).

### 2.5. 16S rRNA gene high-throughput sequencing

Soil bacterial communities were analyzed by next-generation amplicon sequencing of 16S rRNA genes. PCR was used to amplify the V4 hypervariable region of 16S rRNA gene in bacteria and archaea with primer 515F (5′-GTGCCAGCMGCCGCGGTAA-3′) and the reverse primer 806R (5′-GGACTACHVGGGTWTCTAAT-3′). PCR mixture (50 μl) consists of 10 μl DNA template, 3 μl each primer, 6 μl nuclease-free PCR-grade water, 3 μl DMSO and 25 μl Phusion High-Fidelity PCR Master Mix, and the PCR amplification process was as follows: initial denaturation at 98°C for 30 s, followed by 30 cycles consisting of denaturation at 98°C for 15 s, annealing at 58°C for 15 s, and extension at 72°C for 15 s. The PCR system was finally kept at 72°C for 1 min. PCR products were purified and subjected on Illumina HiSeq4000 platform of GUHE INFO (Hangzhou, China). High-quality clean reads were obtained by qualitative filtering of the original reads, with sequences with a similarity of 97% clustered into the same Operational Taxonomic Units (OTUs) (Edgar, 2013).

### 2.6. Physicochemical analysis of soils

The content of organic matter (OM), total nitrogen (TN), total phosphorus (TP) and pH of the soil samples were determined according to the previous studies and the national standard methods (Han et al., 2018; Cheng et al., 2019; Mu et al., 2022). The OM in paddy soil was determined by dichromate oxidation and titration with ferrous sulfate solution. The TN and TP in soil were determined by Semi-micro Kjeldahl Method and spectrophotometry. pH was determined with a pH meter (in a 1:5 soil-water ratio suspension).

### 2.7. Statistical analysis

The abundance and standard deviation of all genes were calculated by Microsoft Excel 2010. SPSS 22.0 was used for correlation analysis and ANOVA analysis, and *p <* 0.05 was taken as the significant difference level. Histogram, heat map and boxplot plots were drawn using Origin 2021 software, and RDA analysis was performed using Canoco5. Structural equation models (SEMs) was established using R 4.2.2 with the “piecewiseSEM” package to describe the relationship among fertilization treatment, soil properties, bacterial community, mobile genetic elements (MGEs) and the abundance of ARGs in paddy-upland soil (Lefcheck, 2016). We used the Fisher’s C test to judge the goodness of the modeling results. The models were modified stepwise according to the pathway significance (*p* < 0.05) and the goodness of the model (0 ≤ Fisher’s C/df ≤ 2 and 0.05 < *p* ≤ 1.00).

## 3. Results and discussion

### 3.1. Occurrence patterns of common ARGs and high-risk ARGs in paddy-upland rotation soil

**Figure 1:**
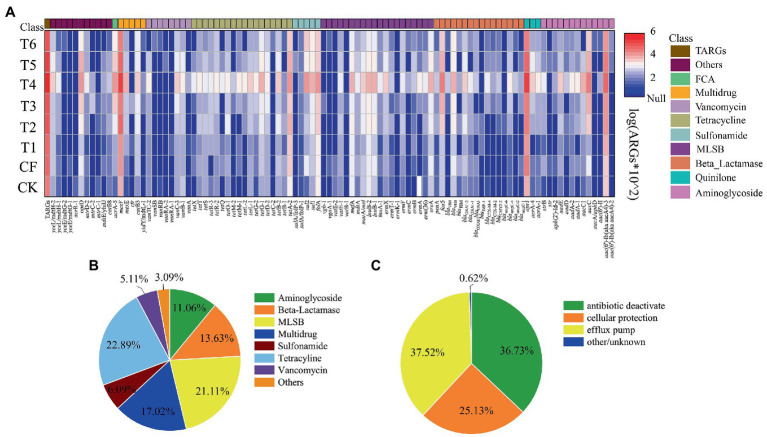
The content of detected ARGs under different fertilization modes in high-throughput sequencing technology **(A)** the abundance of all genes detected, **(B)** proportion of different subtypes of ARGs, **(C)** proportion of antibiotic resistance mechanisms. CK, no fertilization; CF, conventional fertilization; T1, conventional fertilization was reduced by 20%; T2, organic fertilizer replaced T1 (in terms of nitrogen); T3, organic fertilizer replaced T1 and urea supplemented nitrogen (in terms of phosphorus); T4, considering the mineralization rate of organic fertilizer is 25%, organic fertilizer replaced T1 (in terms of nitrogen); T5, considering the mineralization rate of organic fertilizer is 25%, organic fertilizer replaces T1, and urea supplemented nitrogen (in terms of phosphorus); T6, slow release fertilizer.

A total of 100 ARGs subtypes and 9 MGEs (7 transposon genes and 2 integron genes) were detected in paddy-upland rotation soils by HT-qPCR assay (Figure 1A). From the perspective of different categories and mechanisms of action, the ARGs detected in paddy-upland rotation soils included the various types of ARGs (*acc*-ARGs, *bla*-ARGs, *sul*-ARGs, MLSB-ARGs, MDR-ARGs, *tet*-ARGs, *van*-ARGs and other ARGs; Figure 1B). Among them, *tet*-ARGs and MLSB-ARGs were more detected in paddy-upland rotation soil (22.9 and 21.1%, respectively). MDR-ARGs and *bla*-ARGs account for 17.0 and 13.6%, which were high risk for the treatment of human diseases because they could endow bacteria with multidrug resistance, and have been reported in soil by many studies (Chen et al., 2016; Xie et al., 2018; Li et al., 2019). *acc*-ARGs accounted for 11.1%, *sul*-ARGs and *van*-ARGs accounted for 6.1 and 5.1%, respectively. Detected ARGs were classed into four resistance mechanisms in all treatments, including antibiotic deactivation, efflux pump, cellular protection and others, in which antibiotic deactivation and efflux pump (accounting for 36.7 and 37.5%, respectively) were the two dominant mechanisms (Figure 1C). As for the abundance of ARGs in paddy-upland rotation soils, the abundance of MDR-ARGs was the highest, and the sum of all samples was 3.34 × 10^5^ copies, among which the *mex*F abundance of each subtype was the highest. Secondly, *sul*-ARGs, *tet*-ARGs and *bla*-ARGs was more abundant. The total abundance of all treatments with fertilizer application ranged from 6.8 × 10^4^ to 9.6 × 10^4^ copies/g, organic fertilizer application from 1.9 × 10^5^ to 9.8 × 10^5^ copies/g, and slow release fertilizer application from 1.6 × 10^5^ copies/g. Besides, the total abundance of ARGs in organic fertilizer and slow-release fertilizer treatments was also significantly higher than that in unfertilized soil ((4.5 ± 0.6) × 10^4^ copies/g). The above results indicated that agricultural fertilization could increase the pollution load of ARGs in the farmland soil to different degrees, other students have made similar findings (Rahman et al., 2018; Xu Y. et al., 2021).

**Figure 2:**
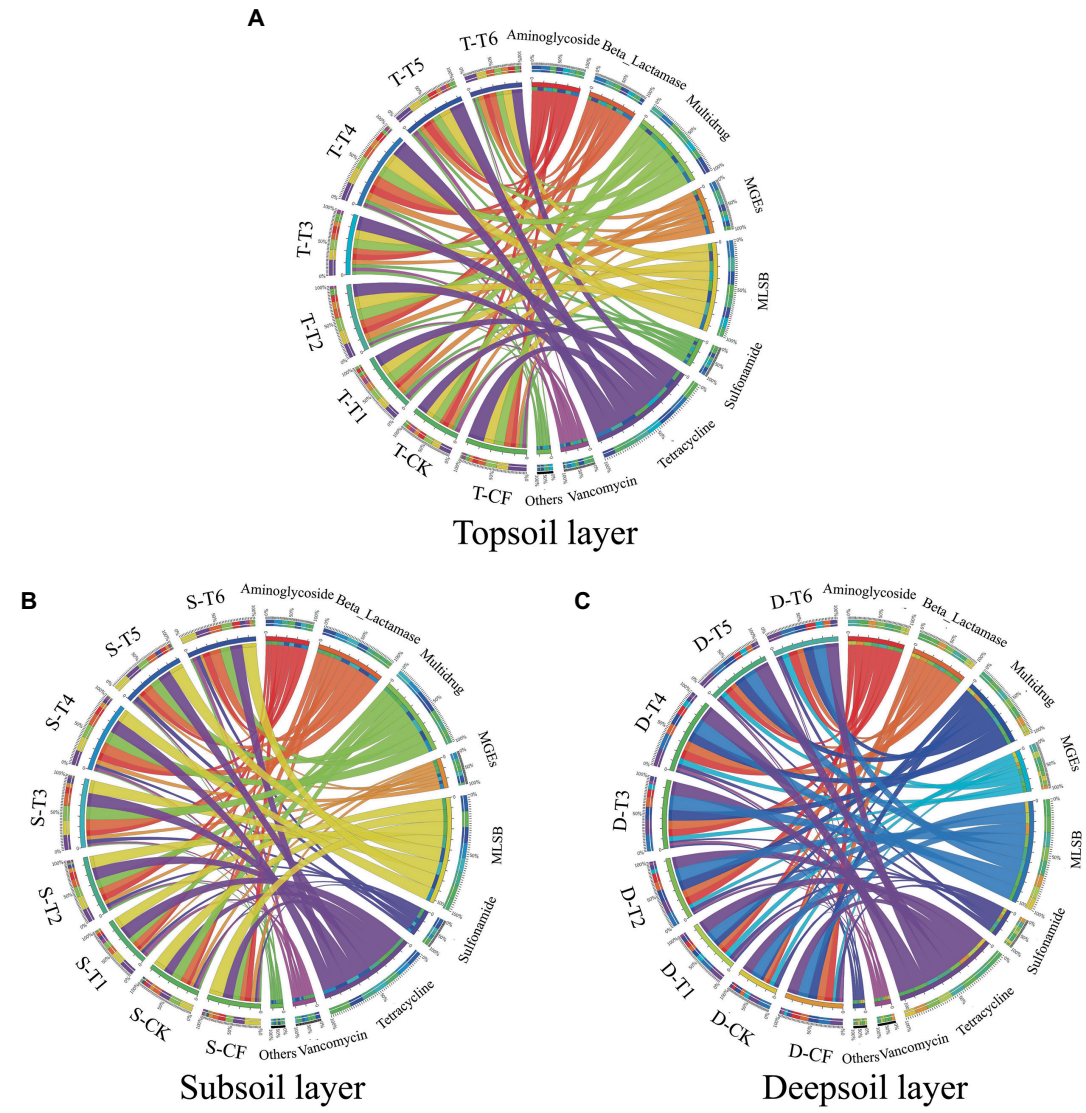
Distribution of each class of ARGs in paddy-upland rotation soil with different fertilization modes and different depths, **(A)** showing 0–20 cm in the topsoil, **(B)** showing 20–50 cm in the subsoil, and **(C)** showing 50–80 cm in the deepsoil. Please refer to Figure 1 for fertilization treatment abbreviations.

It is worth noting that the diversity of ARGs in paddy-upland rotation soil varied with different fertilization modes (Figure 2). There were 68 ARGs subtypes in the paddy-upland rotation soil without fertilization (CK), and 83 ARG subtypes in the T6 soil with slow and controlled release fertilizer. The gene subtypes of ARGs were the most abundant in manured soils of T2, T3, T4 and T5, which was consistent with a previous study, in which the diversity of ARGs increased in the soil environment after application of organic fertilizer (Nolvak et al., 2016; Pu et al., 2020). Moreover, similar to the diversity characteristics of ARGs pollution, MGEs were detected at the largest number in T4 with the largest amount of manure application, which was significantly higher than that in other fertilization types (*p <* 0.05). According to the Venn diagram (Supplementary Figure S1), 53 gene subtypes (48.6%) of ARGs were core ARGs (i.e., co-occurring ARGs) in paddy-upland rotation soils of six different fertilization modes. Among them, the diversity and number of ARGs were the lowest in CK, and the number of ARGs increased in other treatments after fertilization. Notably, mineral fertilizer application also affected ARG abundance to a certain extent, which may be related to changes in soil microbial structure (Sun et al., 2019). The total detected number of ARGs was the highest in the treatment of manure application, followed by the treatment of slow release fertilizer and chemical fertilizer, which were 1.4, 1.3 and 1.1 times higher than that in unfertilized soil, respectively. The diversity of ARGs in soil with organic fertilizer application was more complex than that in unfertilized soil, which significantly changed the composition of ARGs (*p <* 0. 05) and was consistent with previous studies (Zhang et al., 2017; Han et al., 2018; Guo et al., 2022).

### 3.2. Contribution of different fertilization modes to soil ARG abundances

**Figure 3:**
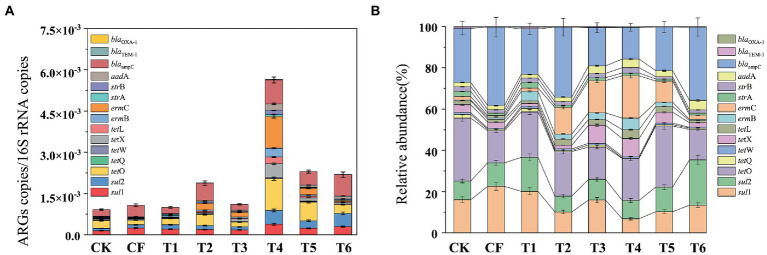
The level **(A)** and the proportion **(B)** in relative abundance of ARGs in paddy-upland rotation soil under different fertilization modes. Please refer to Figure 1 for fertilization treatment abbreviations.

Based on the diversity of ARGs pollution in paddy-upland rotation soils under different fertilization methods, ARGs abundance was calculated to reveal the effect of fertilization modes on antibiotic resistome in soil. Figure 3 showed that the application of chemical fertilizer, organic fertilizer, slow release fertilizer all increased the abundance of ARGs. In paddy-upland rotation soils, the application of chemical fertilizer (T1, T2) increased *str*-ARGs and *erm*-ARGs slightly, while the application of slow-release fertilizer (T6) increased *str*-ARGs and *sul*-ARGs significantly. In addition, the application of commercial organic fertilizer in soil significantly increased the abundance of *tet*-ARGs, *str*-ARGs, *erm*-ARGs and *sul*-ARGs, which were greatly affected by the introduction of these genes into soil. Guo et al. (2018) also reported higher relative abundance of *tet*-ARGs and *sul*-ARGs in manure-amended soils. Additionally, consistent with previous studies, the level of ARG abundance in soil under fertilizer application was similar to that of CK, and the effect of chemical fertilizer on the ARGs abundance of paddy-upland rotation soil was not obvious (Liu et al., 2017; Wang F. H. et al., 2020). The total relative abundance was still the highest in T4, followed by T5 and T6, which were 5.5, 2.3 and 1.2 times of CK, respectively. The order of total relative abundance was T4 > T5 > T6 > T1 > T3 > T2 > CF > CK, the rule of ARGs abundance was slightly different from that of absolute abundance, but the effect of fertilizer application on ARGs abundance in paddy-upland rotation soil was basically that of commercial organic fertilizer > slow release fertilizer > chemical fertilizer.

The proportion of abundance of various ARGs in different fertilization modes were the same, *bla*_ampC_ occupied the highest proportion (the ratio of 15.30–60.01%), followed by *sul*1, *sul*2 and *tet*O, which is closely related to human health, was high in all paddy-upland rotation fields and its distribution was widespread, that should be paid attention to. Although the proportion of *sul*-ARGs (*sul*1 and *sul*2) decreased significantly in commercial organic fertilizer application, the abundance of *sul*-ARGs increased by 1.1–2.6 times compared with CK. In a previous study that applied cow manure to agricultural soils, *sul*-ARGs abundance was also significantly increased, with *sul*1 abundance increasing by 36% compared to background soil levels (Munir and Xagoraraki, 2011). This may be related to the fact that sulfonamides were commonly used as feed additives and antibiotics in the aquaculture industry, and since sulfonamides cannot be synthesized by microorganisms, sulfonamide resistance acquired by microorganisms was mainly due to the selective pressure (Guo et al., 2022). The distribution patterns of absolute and relative abundance of manure application treatments were slightly different, which might be caused by the difference of soil microbial biomass in different fertilization modes. In the treatment of chemical fertilizer, organic fertilizer and slow release fertilizer, the proportion of various ARGs changed, which may be due to the key role of host microorganisms in controlling the spread of ARGs in soil (Chen et al., 2019a; Li et al., 2021; Zhang et al., 2023). Overall, application of manure significantly increased the abundance of ARGs and changed the pollution distribution characteristics of different ARGs. The proportion of *sul*2 in T6 treatment with slow and controlled release fertilizer increased significantly compared with other treatments (*p <* 0.05), and the abundance was the highest in T6. Application of slow release fertilizer may have significant effects on *sul*2-related microbial community in soil to a large extent, which needs further study.

### 3.3. Vertical migration of prevalent ARGs in soils under different fertilization modes

The absolute and relative abundances of ARGs showed different patterns in different soil layers under different fertilization treatments (*p* < 0.05). Supplementary Figure S2 showed that the five subtypes detected ARGs accumulated in the surface layer. In general, the absolute abundance of ARGs in paddy-upland rotation soils of different soil layers was the highest in topsoil, and decreased gradually with the increase of soil depth, with a distribution pattern of 0–20 cm > 20–50 cm > 50–80 cm. Moreover, the absolute abundance of *sul*-ARGs decreased most significantly in the subsoil, which decreased by about 1.5 logs compared with the topsoil. The absolute abundance of ARGs decreased in subsoil layer (20–50 cm), probably due to leaching from topsoil to deeper soil (Jechalke et al., 2014). For the relative abundance of ARGs in different soil layers, some categories accumulated in the surface layer, while others enriched in the deep layer, with inconsistent regularity. In terms of the average relative abundance of total ARGs, it generally showed a vertical accumulation to deepsoil, which was the same as the results of previous studies (Mckinney et al., 2018). This may be due to the fact that the paddy-upland rotation soil in this study was collected after harvest, and ARGs gradually migrated to the deeper layer after fertilization period of more than 1 month. In addition, after the flooding period of rice planting for a long time, the bacteria carrying ARGs accelerated their migration to the subsoil by the osmosis of water. In detail, *sul*-ARGs and *tet*-ARGs had the highest relative abundance in topsoil (average relative abundance of both was about 10^−4^), while *erm*-ARGs, *str*-ARGs and *β*-Lactam-ARGs had the highest relative abundance in subsoil (average relative abundance was about 10^−4^, 10^−5^ and 10^−4^). *sul*-ARGs was still higher in topsoil (8.18 × 10^−5^–9.98 × 10^−4^) than in subsoil and deepsoil (1.10 × 10^−4^–5.07 × 10^−4^ and 4.95 × 10^−6^–5.53 × 10^−4^), which may be related to the higher mobility of sulfa antibiotics and the lower soil absorption rate (Jechalke et al., 2014). Meanwhile, due to the uncertainty of soil properties, related MGEs and other factors, the distribution and accumulation of different types of ARGs in different soil layers may be different, with some enriched in 0–20 cm and others mainly in 50–80 cm.

### 3.4. Different fertilization modes altered soil bacterial community composition

**Figure 4:**
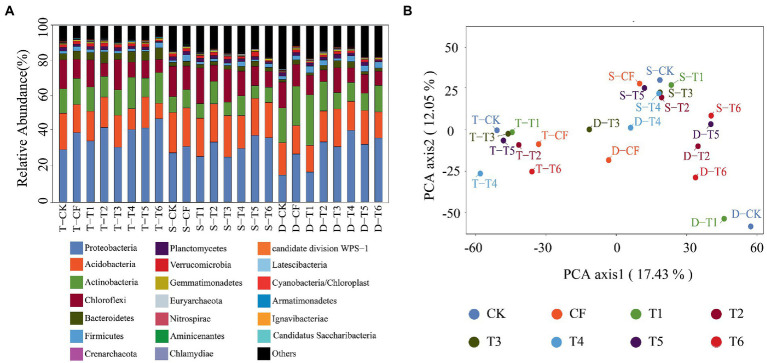
Distribution characteristics of soil microbial community under different fertilization modes, **(A)** showing the abundance of microbial community at each site across different soil layers; **(B)** showing PCA analysis of microbial communities. T. 0–20 cm in the topsoil; S. 20–50 cm in the subsoil, D. 50–80 cm in the deepsoil. Fertilization treatment abbreviation is shown in Figure 1.

The fertilization modes affected soil microbial community dramatically, and the soil microbial community change also occurred in the different dosage. Microorganisms were considered to be the main carriers of ARGs transmission, so the entry of host bacteria of ARGs in manure into the environment would change the local microbial community and promote the migration of ARGs (Li et al., 2020). Since fertilizer application and slow release fertilizer could not support plasmid transfer (Musovic et al., 2014), we supposed that the response of soil microbial community to fertilizer application might be a key factor in the changes of soil ARGs spectrum with inorganic fertilizer application. Over 100,000 sequences were obtained with over 40,000 sequences per sample on average. These sequences were assigned to 6,418 OTUs at 97% similarity. Rarefaction curves for OTUs indicated the sufficient depth of sequencing to account for most of the taxa amplified, ensuring the accuracy of sequencing. Figure 4A showed the compositions of the bacterial communities and the dynamic changes in the bacteria abundances at the top 20 phyla, respectively. The bacterial community compositions of the sample sites were similar at phylum level. *Proteobacteria*, *Acidobacteria*, *Actinobacteria*, *Chloroflexi* and *Bacteriodetes* were the dominant phyla, ranging from 69.6 to 87.6% of the total bacterial community. *Proteobacteria* was the most abundant phyla, accounting for 15.2 to 47.2%, dominated by the classes *Alphaproteobacteria*, *Gammaproteobacteria* and *Betaproteobacteria*. *Acidobacteria* was the second abundant phyla and dominated by *Acidobacteria-Gp6* at class level.

Different fertilization modes changed the proportion of microbial abundance in microbial communities, and the abundance of microorganisms in different soil layers was also different due to the different soil properties. In the top five phylum levels, the proportion of *Proteobacteria*, *Actinobacteria* and *Bacteroidetes* in the soil with chemical fertilizer application (CF and T1) and slow controlled release fertilizer (T6) increased, while the proportion of *Acidobacteria* and *Chloroflexi* decreased. In T4 with the highest ARGs abundance, the proportion of *Proteobacteria* and *Actinobacteria* increased significantly (*p <* 0.05), 1.4 and 1.3 times of CK, respectively. *Actinobacteria* and *Proteobacteria* are considered to be important carriers of ARGs (Stegmann et al., 2015), and their increase could explain that the abundance of ARGs increases with the application of soil commercial organic fertilizer. The changes of ARGs in organic fertilizer application soil may be closely related to these two kinds of microorganisms. Consistent with previous studies (Chaudhry et al., 2012; Zhen et al., 2014), this work found an enhancement of manure and an inhibition of chemical fertilizer on the activity of soil microbial community; but the enhancement of manure was dosage-related. Therefore, the consequence of excessive use of manure involves not only a bloom of soil ARGs but also a suppression of soil microbial activity. The evolution of soil microbial community was significantly affected by the application of different fertilizers, and the composition of soil microbial community was closely related to ARGs migration.

Meanwhile, differences in the composition of microbial communities at different depths may also lead to changes in the abundance of ARGs. According to Principal component analysis (PCA) in Figure 4B, species diversity and microbial community composition of the samples in the same soil layer are similar, but there are differences in microbial community characteristics among different soil layers. The proportion of *Proteobacteria* with the highest abundance decreased significantly from 29.7–47.2% in topsoil to 15.2–40.7% in deepsoil. The effects of different fertilization methods on soil microbial communities at different depths were mainly concentrated in the surface layer, the evolution of microbial communities significantly affected the characteristics of ARGs (Zhang J. Y. et al., 2018).

### 3.5. Impacts of soil properties, bacterial communities and MGEs on ARGs distribution

The characteristics of ARGs in paddy-upland rotation soil under different fertilization modes were affected by many factors. In order to explore the potential factors responsible for the differences in the distribution of ARGs in different fertilizer modes, redundancy analysis was performed to determine the relationship between environmental factors, bacterial community and ARGs in this study, as shown in Supplementary Figure S3. Bacterial communities had an effect on the change of ARGs abundance, and *Proteobacteria*, which had the highest proportion in abundance and changed most obviously with the application of commercial organic fertilizer, was positively correlated with most changes of ARGs. Zhou et al. (2019b) also reported a high proportion of *Proteobacteria* in soil microorganisms applied with manure, and *Proteobacteria* pathogens may obtain ARGs from antibiotic-producing *actinomycetes* through horizontal transfer (Jiang et al., 2017). Soil microbial communities played a key role in controlling the spread of ARG in fertilized soils (Chen et al., 2019a).

**Figure 5:**
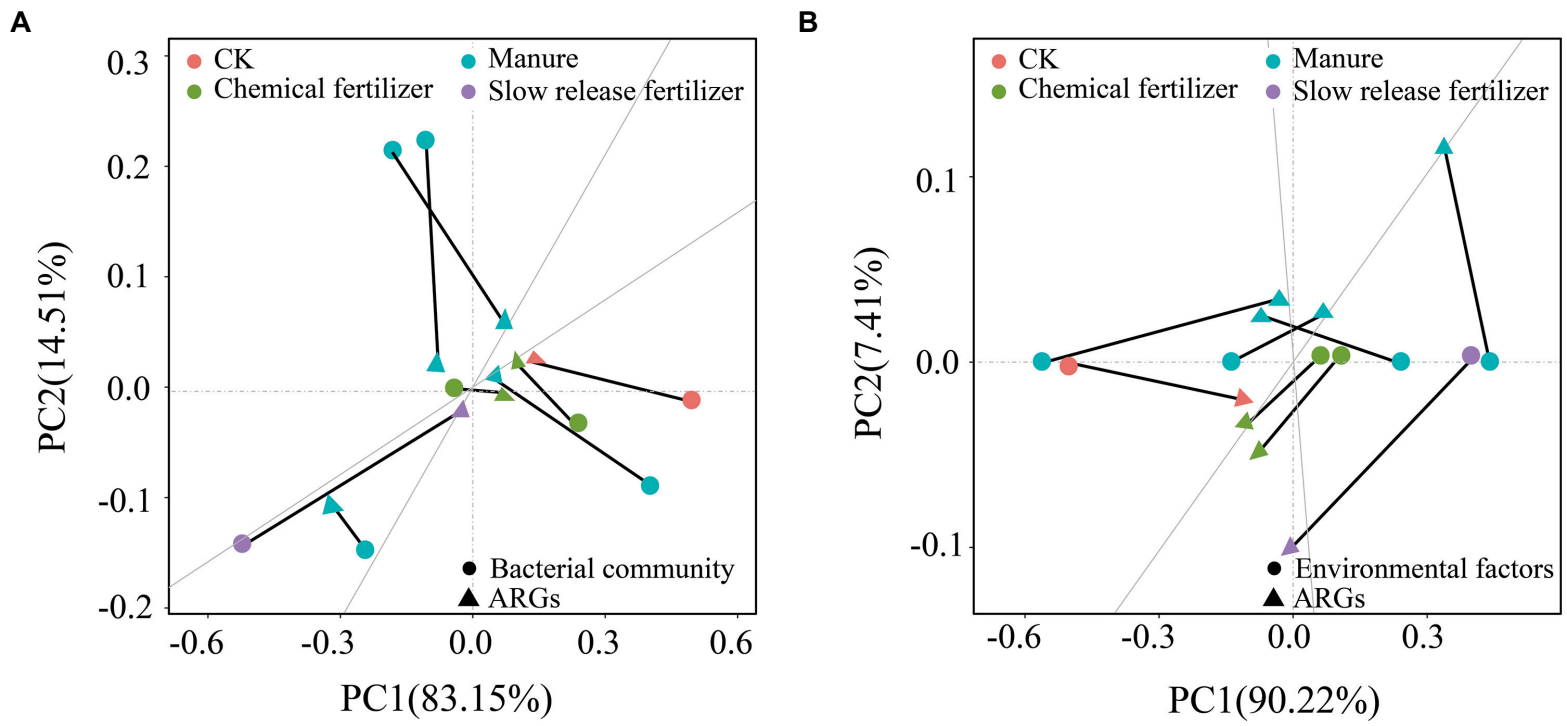
Procrustes analysis (PC) showing the correlation between microbial community **(A)** and environmental factors **(B)** with changes in ARGs in paddy soils under different fertilization modes.

In addition to microbes, changes of environmental factors (pH, TN, TP and OM) were also important indicators for tracking the variation of ARGs. The results showed that OM accounted for 48.1% of ARGs, which was significantly positively correlated with most of ARGs (*p <* 0.05). It was explained that the application of manure increased OM, which significantly affected the distribution characteristics of ARGs. Therefore, the amount of organic fertilizer applied has an impact on the abundance of ARGs, and reducing the application of manure can reduce the risk of ARGs to the soil environment to a certain extent. TN levels also explained the variations in ARGs between different fertilization modes samples, suggesting that fertilizer application may affect the distribution of ARGs in paddy-upland rotation soil, as some ARGs-carrying bacteria may increase due to increased nitrogen levels (Zhou et al., 2017; Qian et al., 2018). Recent studies have also confirmed that TN, TP and pH were important environmental factors driving the evolution of soil microbial community, possibly further leading to the change of ARGs in soil under different fertilization treatments (Cui et al., 2018; Wang F. H. et al., 2020). In addition, all samples were projected by Redundancy analysis (RDA), demonstrating a correlation between bacterial communities, environmental factors and the abundance of ARGs in soils with different fertilization modes. For different fertilization modes, Procrustes analysis (PC) (Figure 5) showed that changes in the abundance of ARGs in soil with fertilizer application were affected by both bacterial community and environmental factors, while different treatments in soil with organic fertilizer application were more concentrated in the environmental factors, and ARGs was mainly dominated by environmental factors.

**Figure 6:**
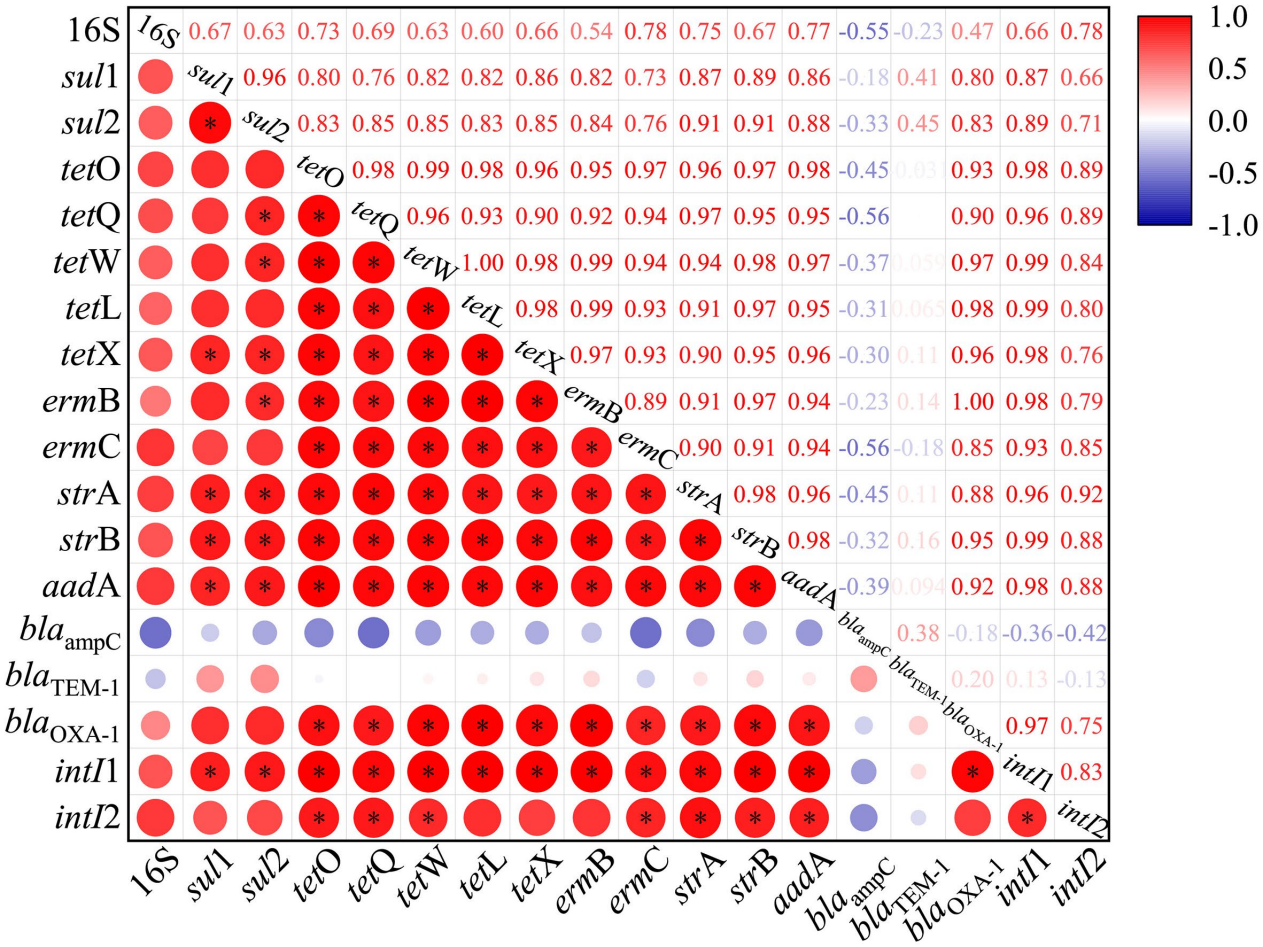
Correlation analysis between ARGs and MGEs in paddy soils, *Correlation is significant (*p* < 0.01).

The transmission and transfer of ARGs among environmental microorganisms are closely related to MEGs (Wang L. et al., 2020), so this study analyzed the correlation between ARGs and MGEs. The results showed that some ARGs were significantly correlated with *int*I1 and *int*I2, and MGEs promoted the migration and propagation of ARGs in paddy-upland rotation soil. The abundance of MGEs was also affected by different fertilization patterns, and its variation pattern was basically consistent with that of ARGs, showing change rule of T4 > T5 > T6 > T2 > T3 > T1 > CF > CK. Figure 6 showed the correlation coefficients between MGEs and the abundance of various ARGs. The results showed that *intI*1 was significantly positively correlated with *tet*-ARGs (*tet*O, *tet*Q, *tet*W, *tet*X and *tet*L), *str*-ARGs (*str*A, *str*B, *aad*A), *erm*B and *bla*_OXA-1_ (*r >* 0.95, *p <* 0.05), *intI*2 was also significantly positively correlated with *str*-ARGs (*str*A, *str*B, *aad*A), *erm*C, *tet*O and *tet*Q (*r >* 0.85, *p <* 0.05), which was consistent with previous findings that *intI*1 in environmental microorganisms is closely related to many ARGs (Guo et al., 2020; Zheng et al., 2020). The strong correlation may be due to the fact that the potential host bacteria of *tet*-ARGs and *erm*-ARGs share the same integrase gene of *intI*1 (Zhu et al., 2013, 2017b; Chen Z. et al., 2019). Compared with the non-fertilized soil, the abundance of *int*I1 in T4, T5 and T6 treatments increased by 14.8, 6.5 and 4.1 times, respectively, indicating that the application of organic fertilizer could promote the spread of ARGs in the soil (Nolvak et al., 2016; Fresia et al., 2019). MGEs were significantly enriched in paddy-upland rotation soil with organic fertilizer application and slow release fertilizer application. The higher abundance of MGEs and the faster horizontal gene transfer of ARGs promoted the further dissemination and evolution of ARGs in paddy-upland rotation soils, and the process interconnected the planetary microbiomes, thus forming a “high way” for the dispersal of ARGs (Zhu et al., 2017a; Zhu L. J. et al., 2019; Wang L. et al., 2020).

**Figure 7:**
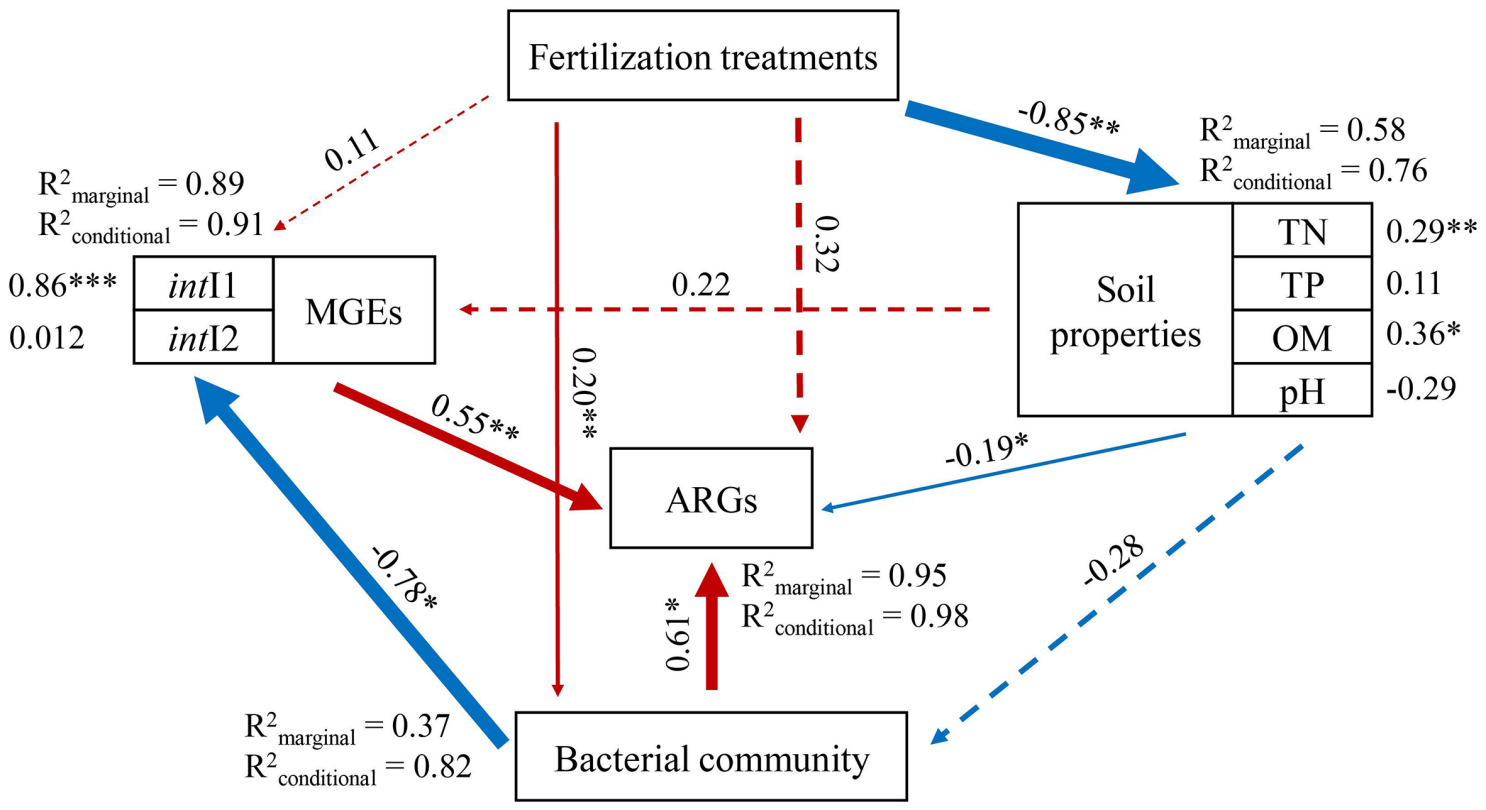
Structural equation models (SEMs) showing the effects of fertilization treatment, soil properties, bacterial community, MGEs and ARGs abundance in paddy-upland soil. Red and blue arrows represent positive and negative relationships, respectively. Solid and dashed arrows denote, respectively, significant and nonsignificant relationships. *R*^2^ represent the proportion of variance explained by all predictors. The hypothetical model fits the data well: Fisher’ C = 19.29, *p* = 0.37, df = 10, AIC = 59.29, BIC = 60.88. Significance levels of each predictor were *p < 0.05, **p < 0.01 and ***p < 0.001.

The relationship between the abundance of ARGs and main driving factors was further depicted by Structural equation models (SEMs) (Figure 7). Different fertilization modes had significant direct effects on soil physicochemical properties (*λ* = −0. 85, *p* < 0.01) and bacterial communities (*λ* = 0. 20, *p* < 0.01), and indirectly affected ARGs abundance. Soil properties had a direct negative effect on the ARGs (*λ* = −0.19, *p* < 0.05) and can also indirectly affect the ARGs by altering bacterial community and MGEs, which both had a positive relationship with the ARGs. Previous studies have shown that TN is associated with efficient lateral transfer of ARGs, with more than 85% of sequenced ARGs and nitrogen processing genes present in the same host bacterial species (Zhang B. et al., 2018; Wang et al., 2021; Peng et al., 2022). Bacterial community directly impacted the abundance of ARGs (*λ* = 0.61, *p* < 0.05) also indirectly influenced MGEs (*λ* = −0.78, *p* < 0.05). MGEs can directly significantly alter the abundance of ARGs (*λ* = 0. 55, *p* < 0.01). *int*I1 gene is one of the most typical integrons and may carry a large number of ARGs fragments, which can be used as an ARGs indicator of influence (Gillings et al., 2015). Therefore, the increase of horizontal transfer potential of ARGs may be closely related to the high abundance of MGEs. Previous studies have shown that horizontal gene transfer plays an important role in ARG abundance under the condition of adequate bacterial community succession nutrition (Zhou et al., 2018; Yao et al., 2020), and in this study, microbial community and MGEs are also two factors that have a great influence on ARGs. Generally, the above analysis results showed that different fertilization modes had indirect effects on the distribution of ARGs by influencing the soil properties, microbial community and MGEs, among which the more significant effects on ARGs were related to the bacterial community of host bacteria and MGEs closely related to horizontal gene transfer.

### 3.6. Environmental implications

The present study, however, has some limitations. First, they fail to reproduce all the factors that might influence the fate of the ARGs. In particular, for paddy-upland rotation soil affected by weather and other natural factors, such as rainfall, drought and flood, previous studies observed soil ARGs migration in previous rainfall simulation experiments (Barrios et al., 2020; Hall et al., 2020). Secondly, the manure used in this study was commercial organic fertilizer refined from cow manure, and the effect of organic fertilizer made from other animal breeds on soil ARGs was not clear. There are some differences in the composition of manure of different animal breeds, and the results may have some influence.

Despite these limitations, ARGs in paddy-upland rotation soils with different fertilization modes had different pollution characteristics based on the results obtained from this study. The application of manure and slow release fertilizer had a greater burden on soil ARGs pollution, and changed the proportion of indigenous microbial community structure in the environment. Moreover, soil properties such as pH, nitrogen and phosphorus also fluctuated with the addition of fertilizers. We suggest to take on the fertilization modes to attention, thus minimizing the dispersal of resistance in soil and to human: (1) The application of manure should be appropriate, and it is best not to provide all the nutrients needed by crops with manure, which is easy to cause excessive nitrogen or phosphorus elements in the soil, and can promote the accumulation of ARGs to some extent; (2) In the same situation, when fertilizers are supplemented to crops, the application of slow release fertilizers should be reduced as far as possible, and simple chemical fertilizers can be used instead.

## 4. Conclusion

This study demonstrated that commercial organic and chemical/slow release fertilization resulted in redistribution of soil ARGs, which was closely related to the structural changes of soil microorganisms in different habitats. Meanwhile, MGEs and environmental factors, like OM and TN, also act as an important driving force in this process. Agricultural fertilization mainly increased the burden of ARGs pollution in topsoil, and some ARGs showed an enrichment trend with the increase of depth. Notably, there was no significant change in the abundance of total soil ARGs under chemical fertilizer at the current applied dose, while slow release fertilizer promoted the propagation of ARGs. These findings highlighted the potential role of fertilization patterns in influencing the fate of ARGs. The combined application of chemical fertilizer and organic fertilizer can provide enough crop nutrients and recycle waste resources to alleviate ARGs pollution and balance soil microbial community, so as to restore the health of soil environment and the stability of ecosystem. Therefore, we believe that the future agricultural fertilization needs to place more emphasis on precise positioning and matching, while the impact on soil ARGs also needs to be monitored and assessed over time.

## Data availability statement

The raw data supporting the conclusions of this article will be made available by the authors, without undue reservation.

## Author contributions

BH: data analysis and writing-original draft preparation. SS: sample pretreatment, DNA extraction, and MGEs detection. FY: supervision and writing-reviewing and editing. XW: ARGs detection and MGEs detection. WG: farm investigation and sample collection. KZ: project administration and supervision. All authors contributed to the article and approved the submitted version.

## Funding

This study was financially supported by National Natural Science Foundation of China (42277033; 42077355; and 42107456), Fundamental Cutting-edge Projects of Research Institute (2022-jcqyrw-dyz), Central Public-interest Scientific Institution Basal Research Fund (No. Y2021PT01), Tianjin Dairy Cattle (mutton sheep) Industry Technology system innovation team construction project (ITTCRS2021000), and Transformation and Promotion Project of Agricultural Science and Technology Achievements in Tianjin (No. 202101040).

## Conflict of interest

The authors declare that the research was conducted in the absence of any commercial or financial relationships that could be construed as a potential conflict of interest.

## Publisher’s note

All claims expressed in this article are solely those of the authors and do not necessarily represent those of their affiliated organizations, or those of the publisher, the editors and the reviewers. Any product that may be evaluated in this article, or claim that may be made by its manufacturer, is not guaranteed or endorsed by the publisher.
