# Supplementary material for: Exploring antibiotic resistance load in paddy-upland rotation fields amended with commercial organic and chemical/slow release fertilizer
Source: Front Microbiol. 2023 Apr 14;14:1184238. doi: 10.3389/fmicb.2023.1184238 (PMC10140351; doi:10.3389/fmicb.2023.1184238)
Supplement: Supplementary file 1 [file Data_Sheet_1.docx]

Supplementary Material

Exploring antibiotic resistance load in paddy-upland rotation fields amended with commercial organic and chemical/slow release fertilizer

Bingjun Han^1^, Shizhou Shen^1,2^, Fengxia Yang^1,2*^, Xiaolong Wang^3^, Wenxuan Gao^1^, Keqiang Zhang^1,2*^

*** Correspondence:** Fengxia Yang: yangfengxiacomeon@163.com (FX. Yang), Keqiang Zhang: keqiangzhang68@163.com (KQ. Zhang)

# Supplementary Figures and Tables

## Supplementary Tables

**Supplementary Table 1.** Fertilization amount of rice, garlic and broad bean under different fertilization treatments

| Treatments | rice | | | | |  | garlic | | | | |  | broad bean | | | |
| --- | --- | --- | --- | --- | --- | --- | --- | --- | --- | --- | --- | --- | --- | --- | --- | --- |
|  | Commercial organic fertilizer  (kg·ha^-1^) | Chemical fertilizer  (kg·ha^-1^) | | | Slow release fertilizer  (kg·ha^-1^) |  | Commercial organic fertilizer  (kg·ha^-1^) | Chemical fertilizer  (kg·ha^-1^) | | | Slow release fertilizer  (kg·ha^-1^) |  | Commercial organic fertilizer  (kg·ha^-1^) | Chemical fertilizer  (kg·ha^-1^) | | Slow release fertilizer  (kg·ha^-1^) |
|  |  | Urea | SSP | SOP |  |  |  | Urea | SSP | SOP |  |  |  | SSP | SOP |  |
| CK | 0 | 0 | 0 | 0 | 0 |  | 0 | 0 | 0 | 0 | 0 |  | 0 | 0 | 0 | 0 |
| CF | 0 | 424 | 450 | 180 | 0 |  | 0 | 1467 | 1125 | 289 | 0 |  | 0 | 480 | 0 | 0 |
| T1 | 0 | 339 | 375 | 144 | 0 |  | 0 | 1174 | 900 | 231 | 0 |  | 0 | 384 | 138 | 0 |
| T2 | 6500 | 0 | 0 | 0 | 0 |  | 22500 | 0 | 0 | 0 | 0 |  | 4150 | 264 | 0 | 0 |
| T3 | 2609 | 203 | 0 | 0 | 0 |  | 6261 | 848 | 0 | 0 | 0 |  | 1250 | 264 | 0 | 0 |
| T4 | 26000 | 0 | 0 | 0 | 0 |  | 90000 | 0 | 0 | 0 | 0 |  | 16600 | 0 | 0 | 0 |
| T5 | 10436 | 203 | 0 | 0 | 0 |  | 25044 | 848 | 0 | 0 | 0 |  | 5000 | 264 | 0 | 0 |
| T6 | 0 | 0 | 0 | 0 | 678 |  | 0 | 0 | 0 | 0 | 2347 |  | 0 | 0 | 0 | 417 |

**Supplementary Table 2.** The HT-qPCR primers used in this study

| **Gene Name** | **Forward Primer** | **Reverse Primer** | **Classification** | **Mechanism** |
| --- | --- | --- | --- | --- |
| *aac*(6')-Ib(aka aacA4)-01 | GTTTGAGAGGCAAGGTACCGTAA | GAATGCCTGGCGTGTTTGA | Aminoglycoside | antibiotic deactivate |
| *aac*(6')-Ib(aka aacA4)-02 | CGTCGCCGAGCAACTTG | CGGTACCTTGCCTCTCAAACC | Aminoglycoside | antibiotic deactivate |
| *aac*(6')-Ib(aka aacA4)-03 | AGAAGCACGCCCGACACTT | GCTCTCCATTCAGCATTGCA | Aminoglycoside | antibiotic deactivate |
| *aac*(6')-II | CGACCCGACTCCGAACAA | GCACGAATCCTGCCTTCTCA | Aminoglycoside | antibiotic deactivate |
| *aac*(6')-Iy | GCTTTGCGGATGCCTCAAT | GGAGAACAAAAATACCTTCAAGGAAA | Aminoglycoside | antibiotic deactivate |
| *aac*A/*aph*D | AGAGCCTTGGGAAGATGAAGTTT | TTGATCCATACCATAGACTATCTCATCA | Aminoglycoside | antibiotic deactivate |
| *aac*C | CGTCACTTATTCGATGCCCTTAC | GTCGGGCGCGGCATA | Aminoglycoside | antibiotic deactivate |
| *aac*C1 | GGTCGTGAGTTCGGAGACGTA | GCAAGTTCCCGAGGTAATCG | Aminoglycoside | antibiotic deactivate |
| *aac*C2 | ACGGCATTCTCGATTGCTTT | CCGAGCTTCACGTAAGCATTT | Aminoglycoside | antibiotic deactivate |
| *aac*C4 | CGGCGTGGGACACGAT | AGGGAACCTTTGCCATCAACT | Aminoglycoside | antibiotic deactivate |
| *aad*A-01 | GTTGTGCACGACGACATCATT | GGCTCGAAGATACCTGCAAGAA | Aminoglycoside | antibiotic deactivate |
| *aad*A-02 | CGAGATTCTCCGCGCTGTA | GCTGCCATTCTCCAAATTGC | Aminoglycoside | antibiotic deactivate |
| *aad*D | CCGACAACATTTCTACCATCCTT | ACCGAAGCGCTCGTCGTATA | Aminoglycoside | antibiotic deactivate |
| *aad*E | TACCTTATTGCCCTTGGAAGAGTTA | GGAACTATGTCCCTTTTAATTCTACAATCT | Aminoglycoside | antibiotic deactivate |
| *acr*A-01 | CAACGATCGGACGGGTTTC | TGGCGATGCCACCGTACT | (flouro)quinilone | efflux pump |
| *acr*A-04 | TACTTTGCGCGCCATCTTC | CGTGCGCGAACGAACAT | (flouro)quinilone | efflux pump |
| *acr*A-05 | CGTGCGCGAACGAACA | ACTTTGCGCGCCATCTTC | FCA | efflux pump |
| *acr*R-01 | GCGCTGGAGACACGACAAC | GCCTTGCTGCGAGAACAAA | other/efflux | efflux pump |
| *acr*R-02 | GATGATACCCCCTGCTGTGAGA | ACCAAACAAGAAGCGCAAGAA | other/efflux | efflux pump |
| *bla_amp_*_C-01_ | TGGCGTATCGGGTCAATGT | CTCCACGGGCCAGTTGAG | Beta_Lactamase | antibiotic deactivate |
| *bla_amp_*_C-02_ | GCAGCACGCCCCGTAA | TGTACCCATGATGCGCGTACT | Beta_Lactamase | antibiotic deactivate |
| *bla_amp_*_C-04_ | TCCGGTGACGCGACAGA | CAGCACGCCGGTGAAAGT | Beta_Lactamase | antibiotic deactivate |
| *bla_amp_*_C-06_ | CCGCTCAAGCTGGACCATAC | CCATATCCTGCACGTTGGTTT | Beta_Lactamase | antibiotic deactivate |
| *aph* | TTTCAGCAAGTGGATCATGTTAAAAT | CCAAGCTGTTTCCACTGTTTTTC | Aminoglycoside | antibiotic deactivate |
| *aph*(2')-Id-01 | TGAGCAGTATCATAAGTTGAGTGAAAAG | GACAGAACAATCAATCTCTATGGAATG | Aminoglycoside | antibiotic deactivate |
| *aph*(2')-Id-02 | TAAGGATATACCGACAGTTTTGGAAA | TTTAATCCCTCTTCATACCAATCCATA | Aminoglycoside | antibiotic deactivate |
| *bla*_ACC-1_ | CACACAGCTGATGGCTTATCTAAAA | AATAAACGCGATGGGTTCCA | Beta_Lactamase | antibiotic deactivate |
| *Bla* | AAAGCCTCAT GGGTGCATAAA | ATAGCTTTTGTTTGCCAGCATCA | Beta_Lactamase | antibiotic deactivate |
| *bla*_CMY2-02_ | GCGAGCAGCCTGAAGCA | CGGATGGGCTTGTCCTCTT | Beta_Lactamase | antibiotic deactivate |
| *bla*_CTX-M-02_ | GCCGCGGTGCTGAAGA | ATCGGATTATAGTTAACCAGGTCAGATTT | Beta_Lactamase | antibiotic deactivate |
| *bla*_CTX-M-04_ | CTTGGCGTTGCGCTGAT | CGTTCATCGGCACGGTAGA | Beta_Lactamase | antibiotic deactivate |
| *bla*_CTX-M-05_ | GCGATAACGTGGCGATGAAT | GTCGAGACGGAACGTTTCGT | Beta_Lactamase | antibiotic deactivate |
| *bla*_IMP-01_ | AACACGGTTTGGTGGTTCTTGTA | GCGCTCCACAAACCAATTG | Beta_Lactamase | antibiotic deactivate |
| *bla*_OXA1_/*bla*_OXA30_ | CGGATGGTTTGAAGGGTTTATTAT | TCTTGGCTTTTATGCTTGATGTTAA | Beta_Lactamase | antibiotic deactivate |
| *bla*_OXA10-01_ | CGCAATTATCGGCCTAGAAACT | TTGGCTTTCCGTCCCATTT | Beta_Lactamase | antibiotic deactivate |
| *bla*_OXA10-02_ | CGCAATTATCGGCCTAGAAACT | TTGGCTTTCCGTCCCATTT | Beta_Lactamase | antibiotic deactivate |
| *bla*_ROB_ | GCAAAGGCATGACGATTGC | CGCGCTGTTGTCGCTAAA | Beta_Lactamase | antibiotic deactivate |
| *bla*_SHV-01_ | TCCCATGATGAGCACCTTTAAA | TTCGTCACCGGCATCCA | Beta_Lactamase | antibiotic deactivate |
| *bla*_TEM_ | AGCATCTTACGGATGGCATGA | TCCTCCGATCGTTGTCAGAAGT | Beta_Lactamase | antibiotic deactivate |
| *bla*_TLA_ | ACACTTTGCCATTGCTGTTTATGT | TGCAAATTTCGGCAATAATCTTT | Beta_Lactamase | antibiotic deactivate |
| *bla*_VEB_ | CCCGATGCAAAGCGTTATG | GAAAGATTCCCTTTATCTATCTCAGACAA | Beta_Lactamase | antibiotic deactivate |
| *bla*_VIM_ | GCACTTCTCGCGGAGATTG | CGACGGTGATGCGTACGTT | Beta_Lactamase | antibiotic deactivate |
| *cat*B3 | GCACTCGATGCCTTCCAAAA | AGAGCCGATCCAAACGTCAT | (flor)/(chlor)/(am)phenicol | antibiotic deactivate |
| *cat*B8 | CACTCGACGCCTTCCAAAG | CCGAGCCTATCCAGACATCATT | other/efflux | antibiotic deactivate |
| *cfr* | GCAAAATTCAGAGCAAGTTACGAA | AAAATGACTCCCAACCTGCTTTAT | (flor)/(chlor)/(am)phenicol | antibiotic deactivate |
| *cfx*A | TCATTCCTCGTTCAAGTTTTCAGA | TGCAGCACCAAGAGGAGATGT | Beta_Lactamase | antibiotic deactivate |
| *cInt*I-1(class1) | GGCATCCAAGCAGCAAG | AAGCAGACTTGACCTGA | integron | integrase |
| *ere*A | CCTGTGGTACGGAGAATTCATGT | ACCGCATTCGCTTTGCTT | MLSB | antibiotic deactivate |
| *ere*B | GCTTTATTTCAGGAGGCGGAAT | TTTTAAATGCCACAGCACAGAATC | other/efflux | antibiotic deactivate |
| *erm*(34) | GCGCGTTGACGACGATTT | TGGTCATACTCGACGGCTAGAAC | MLSB | cellular protection |
| *erm*(35) | TTGAAAACGATGTTGCATTAAGTCA | TCTATAATCACAACTAACCACTTGAACGT | MLSB | cellular protection |
| *erm*(36) | GGCGGACCGACTTGCAT | TCTGCGTTGACGACGGTTAC | MLSB | cellular protection |
| *erm*A | TTGAGAAGGGATTTGCGAAAAG | ATATCCATCTCCACCATTAATAGTAAACC | MLSB | cellular protection |
| *erm*B | TAAAGGGCATTTAACGACGAAACT | TTTATACCTCTGTTTGTTAGGGAATTGAA | MLSB | cellular protection |
| *erm*C | TTTGAAATCGGCTCAGGAAAA | ATGGTCTATTTCAATGGCAGTTACG | MLSB | cellular protection |
| *erm*F | CAGCTTTGGTTGAACATTTACGAA | AAATTCCTAAAATCACAACCGACAA | MLSB | cellular protection |
| *erm*K-01 | GTTTGATATTGGCATTGTCAGAGAAA | ACCATTGCCGAGTCCACTTT | MLSB | cellular protection |
| *erm*T-01 | GTTCACTAGCACTATTTTTAATGACAGAAGT | GAAGGGTGTCTTTTTAATACAATTAACGA | MLSB | cellular protection |
| *erm*X | GCTCAGTGGTCCCCATGGT | ATCCCCCCGTCAACGTTT | MLSB | cellular protection |
| *erm*Y | TTGTCTTTGAAAGTGAAGCAACAGT | TAACGCTAGAGAACGATTTGTATTGAG | MLSB | cellular protection |
| *fol*A | CGAGCAGTTCCTGCCAAAG | CCCAGTCATCCGGTTCATAATC | Sulfonamide | antibiotic deactivate |
| *fox*5 | GGTTTGCCGCTGCAGTTC | GCGGCCAGGTGACCAA | Beta_Lactamase | antibiotic deactivate |
| *int*I-1(clinic) | CGAACGAGTGGCGGAGGGTG | TACCCGAGAGCTTGGCACCCA | integron | integrase |
| *lnu*A-01 | TGACGCTCAACACACTCAAAAA | TTCATGCTTAAGTTCCATACGTGAA | MLSB | antibiotic deactivate |
| *lnu*B-01 | TGAACATAATCCCCTCGTTTAAAGAT | TAATTGCCCTGTTTCATCGTAAATAA | MLSB | antibiotic deactivate |
| *lnu*B-02 | AAAGGAGAAGGTGACCAATACTCTGA | GGAGCTACGTCAAACAACCAGTT | MLSB | antibiotic deactivate |
| *lnu*C | TGGTCAATATAACAGATGTAAACCAGATTT | CACCCCAGCCACCATCAA | MLSB | antibiotic deactivate |
| *mat*A/*mel* | TAGTAGGCAAGCTCGGTGTTGA | CCTGTGCTATTTTAAGCCTTGTTTCT | MLSB | efflux pump |
| *mdt*A | CCTAACGGGCGTGACTTCA | TTCACCTGTTTCAAGGGTCAAA | MLSB | efflux pump |
| *mdt*E/*yhi*U | CGTCGGCGCACTCGTT | TCCAGACGTTGTACGGTAACCA | other/efflux | efflux pump |
| *mef*A | CCGTAGCATTGGAACAGCTTTT | AAACGGAGTATAAGAGTGCTGCAA | MLSB | efflux pump |
| *mex*E | GGTCAGCACCGACAAGGTCTAC | AGCTCGACGTACTTGAGGAACAC | (flor)/(chlor)/(am)phenicol | efflux pump |
| *mex*F | CCGCGAGAAGGCCAAGA | TTGAGTTCGGCGGTGATGA | (flor)/(chlor)/(am)phenicol | efflux pump |
| *mtr*C-01 | GGACGGGAAGATGGTCCAA | CGTAGCGTTCCGGTTCGAT | other/efflux | efflux pump |
| *mtr*C-02 | CGGAGTCCATCGACCATTTG | ATCGTCGGCAAGGAGAATCA | other/efflux | efflux pump |
| *mtr*D-02 | GGTCGGCACGCTCTTGTC | TGAAGAATTTGCGCACCACTAC | other/efflux | efflux pump |
| *mtr*D-03 | CCGCCAAGCCGATATAGACA | GGCCGGGTTGCCAAA | other/efflux | efflux pump |
| *opr*D | ATGAAGTGGAGCGCCATTG | GGCCACGGCGAACTGA | other/efflux | efflux pump |
| *opr*J | ACGAGAGTGGCGTCGACAA | AAGGCGATCTCGTTGAGGAA | (flouro)quinilone | efflux pump |
| *pbp* | CCGGTGCCATTGGTTTAGA | AAAATAGCCGCCCCAAGATT | Beta_Lactamase | cellular protection |
| *pen*A | AGACGGTAACGTATAACTTTTTGAAAGA | GCGTGTAGCCGGCAATG | Beta_Lactamase | cellular protection |
| *spc*N-01 | AAAAGTTCGATGAAACACGCCTAT | TCCAGTGGTAGTCCCCGAATC | Aminoglycoside | antibiotic deactivate |
| *spc*N-02 | CAGAATCTTCCTGAAAAGTTTGATGAA | CGCAGACACGCCGAATC | Aminoglycoside | antibiotic deactivate |
| *str* | AATGAGTTTTGGAGTGTCTCAACGTA | AATCAAAACCCCTATTAAAGCCAAT | Aminoglycoside | antibiotic deactivate |
| *str*A | CCGGTGGCATTTGAGAAAAA | GTGGCTCAACCTGCGAAAAG | Aminoglycoside | antibiotic deactivate |
| *str*B | GCTCGGTCGTGAGAACAATCT | CAATTTCGGTCGCCTGGTAGT | Aminoglycoside | antibiotic deactivate |
| *sul*1 | CAGCGCTATGCGCTCAAG | ATCCCGCTGCGCTGAGT | Sulfonamide | cellular protection |
| *sul*2 | TCATCTGCCAAACTCGTCGTTA | GTCAAAGAACGCCGCAATGT | Sulfonamide | cellular protection |
| *sul*A/*fol*P-01 | CAGGCTCGTAAATTGATAGCAGAAG | CTTTCCTTGCGAATCGCTTT | Sulfonamide | cellular protection |
| *sul*A/*fol*P-03 | CACGGCTTCGGCTCATGT | TGCCATCCTGTGACTAGCTACGT | Sulfonamide | cellular protection |
| *tet*A-02 | CTCACCAGCCTGACCTCGAT | CACGTTGTTATAGAAGCCGCATAG | Tetracycline | efflux pump |
| *tet*B-01 | AGTGCGCTTTGGATGCTGTA | AGCCCCAGTAGCTCCTGTGA | Tetracycline | efflux pump |
| *tet*B-02 | GCCCAGTGCTGTTGTTGTCAT | TGAAAGCAAACGGCCTAAATACA | Tetracycline | efflux pump |
| *tet*C-01 | CATATCGCAATACATGCGAAAAA | AAAGCCGCGGTAAATAGCAA | Tetracycline | efflux pump |
| *tet*C-02 | ACTGGTAAGGTAAACGCCATTGTC | ATGCATAAACCAGCCATTGAGTAAG | Tetracycline | efflux pump |
| *tet*D-02 | TGTCATCGCGCTGGTGATT | CATCCGCTTCCGGGAGAT | Tetracycline | efflux pump |
| *tet*G-01 | TCAACCATTGCCGATTCGA | TGGCCCGGCAATCATG | Tetracycline | efflux pump |
| *tet*G-02 | CATCAGCGCCGGTCTTATG | CCCCATGTAGCCGAACCA | Tetracycline | efflux pump |
| *tet*H | TTTGGGTCATCTTACCAGCATTAA | TTGCGCATTATCATCGACAGA | Tetracycline | efflux pump |
| *tet*K | CAGCAGTCATTGGAAAATTATCTGATTATA | CCTTGTACTAACCTACCAAAAATCAAAATA | Tetracycline | efflux pump |
| *tet*L-01 | AGCCCGATTTATTCAAGGAATTG | CAAATGCTTTCCCCCTGTTCT | Tetracycline | efflux pump |
| *tet*L-02 | ATGGTTGTAGTTGCGCGCTATAT | ATCGCTGGACCGACTCCTT | Tetracycline | efflux pump |
| *tet*M-01 | CATCATAGACACGCCAGGACATAT | CGCCATCTTTTGCAGAAATCA | Tetracycline | cellular protection |
| *tet*M-02 | TAATATTGGAGTTTTAGCTCATGTTGATG | CCTCTCTGACGTTCTAAAAGCGTATTAT | Tetracycline | cellular protection |
| *tet*O-01 | ATGTGGATACTACAACGCATGAGATT | TGCCTCCACATGATATTTTTCCT | Tetracycline | cellular protection |
| *tet*Q | CGCCTCAGAAGTAAGTTCATACACTAAG | TCGTTCATGCGGATATTATCAGAAT | Tetracycline | cellular protection |
| *tet*R-02 | CGCGATAGACGCCTTCGA | TCCTGACAACGAGCCTCCTT | Tetracycline | efflux pump |
| *tet*R-03 | CGCGATGGAGCAAAAGTACAT | AGTGAAAAACCTTGTTGGCATAAAA | Tetracycline | efflux pump |
| *tet*S | TTAAGGACAAACTTTCTGACGACATC | TGTCTCCCATTGTTCTGGTTCA | Tetracycline | cellular protection |
| *tet*T | CCATATAGAGGTTCCACCAAATCC | TGACCCTATTGGTAGTGGTTCTATTG | Tetracycline | cellular protection |
| *tet*V | GCGGGAACGACGATGTATATC | CCGCTATCTCACGACCATGAT | Tetracycline | efflux pump |
| *tet*X | AAATTTGTTACCGACACGGAAGTT | CATAGCTGAAAAAATCCAGGACAGTT | Tetracycline | other/unknown |
| *tnp*A-01 | CATCATCGGACGGACAGAATT | GTCGGAGATGTGGGTGTAGAAAGT | other | transposase |
| *tnp*A-02 | GGGCGGGTCGATTGAAA | GTGGGCGGGATCTGCTT | other | transposase |
| *tnp*A-03 | AATTGATGCGGACGGCTTAA | TCACCAAACTGTTTATGGAGTCGTT | other | transposase |
| *tnp*A-04 | CCGATCACGGAAAGCTCAAG | GGCTCGCATGACTTCGAATC | other | transposase |
| *tnp*A-05 | GCCGCACTGTCGATTTTTATC | GCGGGATCTGCCACTTCTT | other | transposase |
| *tnp*A-07 | GAAACCGATGCTACAATATCCAATTT | CAGCACCGTTTGCAGTGTAAG | other | transposase |
| Tp614 | GGAAATCAACGGCATCCAGTT | CATCCATGCGCTTTTGTCTCT | other | transposase |
| *van*A | AAAAGGCTCTGAAAACGCAGTTAT | CGGCCGTTATCTTGTAAAAACAT | Vancomycin | cellular protection |
| *van*B-01 | TTGTCGGCGAAGTGGATCA | AGCCTTTTTCCGGCTCGTT | Vancomycin | cellular protection |
| *van*C-01 | ACAGGGATTGGCTATGAACCAT | TGACTGGCGATGATTTGACTATG | Vancomycin | cellular protection |
| *van*C-03 | AAATCAATACTATGCCGGGCTTT | CCGACCGCTGCCATCA | Vancomycin | cellular protection |
| *van*C1 | AGGCGATAGCGGGTATTGAA | CAATCGTCAATTGCTCATTTCC | Vancomycin | cellular protection |
| *van*RA-01 | CCCTTACTCCCACCGAGTTTT | TTCGTCGCCCCATATCTCAT | Vancomycin | cellular protection |
| *van*RA-02 | CCACTCCGGCCTTGTCATT | GCTAACCACATTCCCCTTGTTTT | Vancomycin | cellular protection |
| *van*RB | GCCCTGTCGGATGACGAA | TTACATAGTCGTCTGCCTCTGCAT | Vancomycin | cellular protection |
| *van*SB | GCGCGGCAAATGACAAC | TTTGCCATTTTATTCGCACTGT | Vancomycin | cellular protection |
| *van*TC-01 | CACACGCATTTTTTCCCATCTAG | CAGCCAACAGATCATCAAAACAA | Vancomycin | cellular protection |
| *van*TC-02 | ACAGTTGCCGCTGGTGAAG | CGTGGCTGGTCGATCAAAA | Vancomycin | cellular protection |
| *vat*B-01 | GGAAAAAGCAACTCCATCTCTTGA | TCCTGGCATAACAGTAACATTCTGA | MLSB | antibiotic deactivate |
| *vat*E-01 | GGTGCCATTATCGGAGCAAAT | TTGGATTGCCACCGACAAT | MLSB | antibiotic deactivate |
| *vat*E-02 | GACCGTCCTACCAGGCGTAA | TTGGATTGCCACCGACAATT | MLSB | antibiotic deactivate |
| *vga*A-01 | CGAGTATTGTGGAAAGCAGCTAGTT | CCCGTACCGTTAGAGCCGATA | MLSB | efflux pump |
| *vga*A-02 | GACGGGTATTGTGGAAAGCAA | TTTCCTGTACCATTAGATCCGATAATT | MLSB | efflux pump |
| *vgb*-01 | AGGGAGGGTATCCATGCAGAT | ACCAAATGCGCCCGTTT | MLSB | antibiotic deactivate |
| *yce*E/*mdt*G-01 | TGGCACAAAATATCTGGCAGTT | TTGTGTGGCGATAAGAGCATTAG | other/efflux | efflux pump |
| *yce*E/*mdt*G-02 | TTATCTGTTTTCTGCTCACCTTCTTTT | GCGTGGTGACAAACAGGCTTA | other/efflux | efflux pump |
| *yce*L/*mdt*H-01 | TCGGGATGGTGGGCAAT | CGATAACCGAGCCGATGTAGA | other/efflux | efflux pump |
| *yce*L/*mdt*H-02 | CGCGTGAAACCTTAAGTGCTT | AGACGGCTAAACCCCATATAGCT | other/efflux | efflux pump |
| *yid*Y/*mdt*L-01 | GCAGTTGCATATCGCCTTCTC | CTTCCCGGCAAACAGCAT | (flor)/(chlor)/(am)phenicol | efflux pump |
| *yid*Y/*mdt*L-02 | TGCTGATCGGGATTCTGATTG | CAGGCGCGACGAACATAAT | (flor)/(chlor)/(am)phenicol | efflux pump |
| *int*I2 | TTATTGCTGGGATTAGGC | ACGGCTACCCTCTGTTATC | integron | integron |

## Supplementary Figures


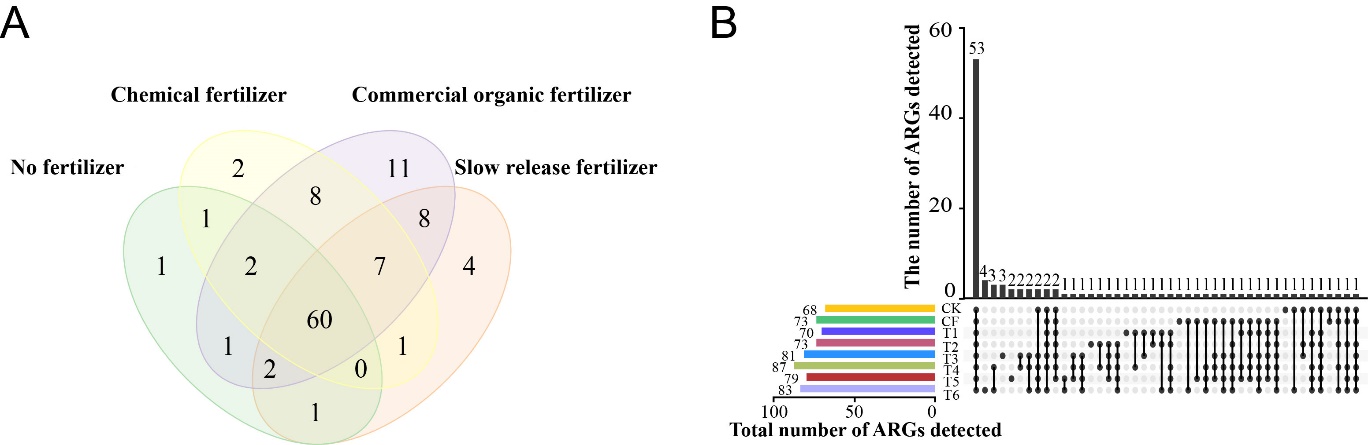


**Supplementary Figure 1.** Venn diagram of ARGs in different fertilization patterns (A) and Upset Venn diagram of ARGs in different treatments (B). Please refer to Figure 1 for fertilization treatment abbreviations.


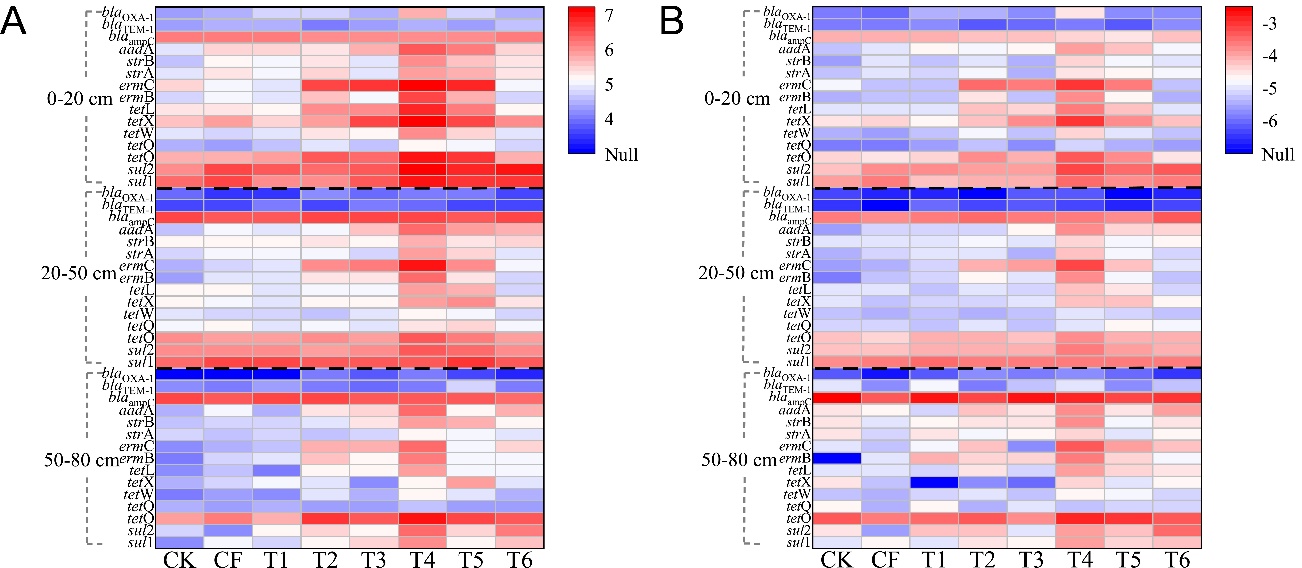


**Supplementary Figure 2.** Changes of different ARGs in different soil layers (0–20 cm in the topsoil, 20–50 cm in the subsoil, 50–80 cm in the deepsoil) (A. the absolute abundance of ARGs, B. the relative abundance of ARGs). Please refer to Figure 1 for fertilization treatment abbreviations.


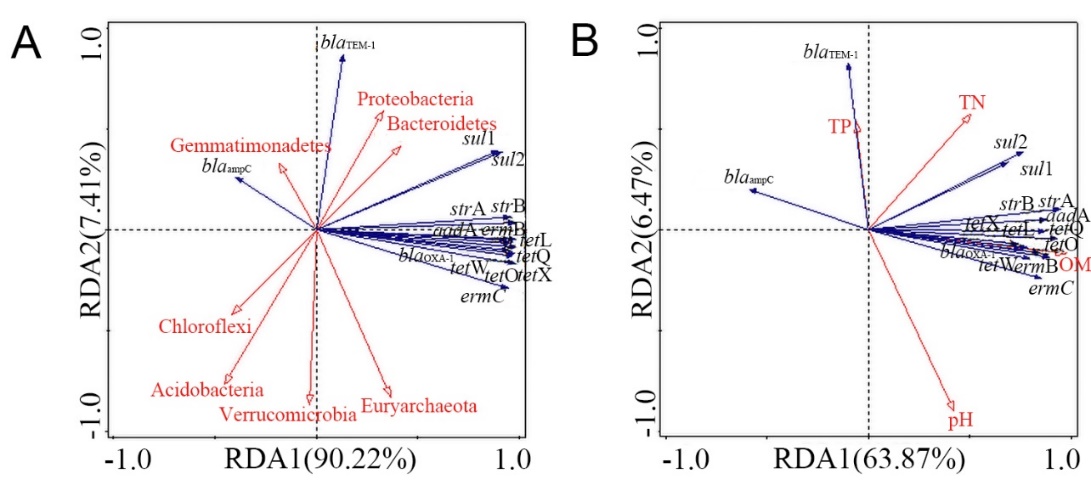


**Supplementary Figure 3.** Redundancy analysis (RDA) of the correlation between ARGs with the top 10 phylum microbial community (A) and environmental factors (B), ARGs by blue lines, and microbial communities and environmental factors are represented by red lines. (Abbreviation: TP, total phosphorus; TN, total nitrogen; OM, organic matter; pH, pH value.)
